# Recent Updates on mRNA Vaccines

**DOI:** 10.3390/vaccines10081209

**Published:** 2022-07-29

**Authors:** Emily Sydow, Abu Salim Mustafa, Asma Hanif, Javed Tunio, Shumaila Nida Muhammad Hanif

**Affiliations:** 1 Department of Basic Sciences, University of Pikeville, Coal Building, Office 718, 147 Sycamore Street, Pikeville, KY 41501, USA; emilysydow@upike.edu; 2 Department of Microbiology, Faculty of Medicine, Kuwait University, Safat 13110, Kuwait; abu.mustafa@ku.edu.kw; 3 Department of Restorative Sciences, College of Dentistry, Kuwait University, Kuwait City 12037, Kuwait; asma.h@hsc.edu.kw; 4 Department of Internal Medicine, Carver College of Medicine, University of Iowa, Iowa City, IA 52242, USA; jtunio@msn.com

Messenger RNA has been studied by everyone, from vaccine developers to high school biology students, since the discovery of its isolation in 1961 [1,2]. However, the COVID-19 pandemic has brought the term “mRNA” into the vernacular of nearly every individual on Earth. The emergent need for vaccination against SARS-CoV-2 was met by the development of three mRNA-based vaccines (CureVac, ClinicalTrials.gov Identifier: NCT04515147; Pfizer-BioNTech, ClinicalTrials.gov Identifier: NCT04368728; and Moderna/NIAID, ClinicalTrials.gov Identifier: NCT04470427) [3,4,5,6,7]. As the public has become vaccinated against COVID-19, questions surrounding the safety, efficacy, and potential of mRNA technology have arisen [8]. While COVID vaccines are the first mRNA-based vaccines to be approved for human use, researchers have been studying their efficacy and safety over the past 60 years in an effort to combat some of medicine’s most challenging disease processes. Understanding how early mRNA vaccine research primed the development of the COVID vaccines allows one to see that mRNA technology will be hugely influential in the management and containment of infectious and non-infectious diseases of the future.

Over the past six decades, researchers have attempted to utilize mRNA to prevent and treat some of the most challenging disease processes. The development of an effective HIV vaccine has eluded vaccine developers and remains a significant challenge to fighting the HIV/AIDS crisis, particularly in developing nations [9]. Due to the rapid mutation rate of the virus, a lack of appropriate animal models, insufficient funding, and lack of information regarding the correlates of immune protection, attempts to manufacture a viable vaccine have been largely unsuccessful. With over 35 million individuals infected each year, prophylactic protection is essential. The tentative success of the HIV-1 mRNA-based vaccine in a phase 1 clinical trial is an exciting development in fighting the spread of HIV [10]. In the phase 1 clinical trial, 97% of participants who received the eOD-GT8 60mer vaccine developed VRC01-class IgG B cells. However, as VRC01 antibodies must mutate to become bNABs, the potential success of the vaccine is still largely unknown [11].

Early research into the potential utilization of mRNA also included cancer treatments. In this issue, readers will find a detailed history of the use of mRNA in cancer treatments in the article “mRNA-Based Vaccines”. In 1995, Conry and coworkers reported protective anti-tumor immunity induced in mice via the intramuscular injection of mRNA encoding carcinoembryonic antigens [12]. Due to the flexibility of mRNA, personalized cancer vaccines can be manufactured to induce humoral and cell-mediated immune responses targeted to tumor-associated antigens (TAAs) [13]. The ability of mRNA vaccines to be manufactured and adjusted quickly is advantageous not only for the treatment of aggressive cancers but also for curtailing the spread of infectious diseases. Without the foundational research provided by cancer and HIV research, the ability of mRNA vaccines to respond to the COVID pandemic would not have been feasible. The COVID-19 pandemic demonstrated the need for rapid vaccine development and global production made possible by mRNA-based vaccines. The Moderna mRNA-1273 vaccine was created within 2 months of the release of the draft viral genome, an unprecedented timeline [5]. Although the Pfizer and Moderna COVID-19 vaccines were the first mRNA-based vaccines to garner FAD approval, successful mRNA-based vaccines for Zika virus, rabies, influenza virus, cytomegalovirus, Ebola virus, *Streptococcus* species, and *Toxoplasma gondii* had been previously studied in animal models. Some of these vaccines are currently being tested clinically but have yet to go beyond early phases. It is important to remember that the speed at which the COVID vaccines were developed was largely influenced by a true global emergency resulting in massive funding. Firms were able to run multiple trials in parallel, and regulators moved at faster speeds than usual [14]. Future vaccines will need similar financial backing and support in order to be developed at the same lightning-fast speed.

Alhough the success of the COVID vaccines provides a promising background for continued mRNA vaccine development, there are still challenges that researchers will need to address in order to apply the technology to other diseases and even future pandemics. At present, adverse reactions to the Pfizer and Moderna COVID vaccines have been primarily limited to hypersensitivity reactions resulting in anaphylaxis and adverse cutaneous reactions [15,16]. Injection site reactions are the most common, and patients with pre-existing chronic inflammatory dermatosis may need to be taken under special consideration when evaluating the best vaccination protocol. Additionally, patients in non-Caucasian ethnic groups may present with different manifestations of ADRs, making diagnosis difficult, and women also appear to experience ADRs post-vaccination at a higher rate than males [17,18]. The phenomenon “COVID-arm” is a localized erythematous rash surrounding the injection site that manifests within days of administering the first dose of the Moderna COVID-19 vaccine [19]. “COVID-arm” has been linked to mRNA-based vaccines, and further studies on the underlying immunological mechanism should be conducted as new mRNA vaccines enter clinical trials [16]. While adverse reactions are rare and are usually self-limiting, typically resolving without therapeutic intervention within a few days, physicians’ education on identifying dermatological conditions needs to be improved in order to provide the best care for patients who experience adverse cutaneous reactions.

While mRNA-based vaccines hint at a more efficient, cost-effective future of vaccine manufacturing, the best delivery system for mRNA vaccines has yet to be determined and remains a significant problem in formulating new vaccines and cancer treatments. Naked mRNA is large and highly negatively charged. Without a carrier, naked mRNA cannot cross the cell membrane to elicit an immunogenic response. However, naked mRNA is stable at room temperatures for months at a time [20]. This provides mRNA with a significant advantage over most vaccines that must be stored and transported in an uninterrupted cold-chain process. The cold-chain process is often unfeasible in rural and tropical areas. It is thus important to develop vectors that allow mRNA to cross the cell membrane without compromising its ability to remain stable at room temperatures. Vectors, including cell-penetrating proteins, lipid carriers, and infected dendritic cells, are currently being studied for their efficacy in clinical trials [21]. The best route of administration also requires further research. Currently, I.M. injection is used as the route of administration of mRNA-based vaccines, but this requires a large volume and may not be ideal in areas where vaccine supply is limited. Intradermal injection has been shown to elicit a similar immunogenic response to I.M. injection while using a significantly smaller dose. As mRNA vaccine research continues, uncovering the best delivery system and route of administration will remain a hot topic.

The past, present, and future of mRNA technology are inherently entangled. Lessons learned from current utilizations will influence ongoing research that laid the foundation for the development of modern vaccines. Looking forward to the present use of mRNA-based vaccines in the COVID pandemic, support and funding for mRNA vaccine research will hopefully be bolstered by the increased worldwide aware-ness of the importance of prophylactic protection in responding to public health crises. Cancer and HIV/AIDS research that laid the groundwork for the development of the COVID vaccines can continue to advance with the knowledge that mRNA-based vaccines are effective and have an excellent safety profile in real-world applications. Physicians’ education will need to improve to best manage and mitigate possible side effects, and patients will need to be counseled in order to increase confidence in mRNA vaccines.

## Data Availability

Not applicable.
